# Potential Side Effects and Adverse Events of Antipsychotic Use for Residents With Dementia in Assisted Living: Implications for Prescribers, Staff, and Families

**DOI:** 10.1177/07334648211023678

**Published:** 2021-06-23

**Authors:** Anna Song Beeber, Sheryl Zimmerman, Christopher J. Wretman, Stephanie Palmertree, Kush Patel, Philip D. Sloane

**Affiliations:** 1 The University of North Carolina at Chapel Hill, USA; 2 Northeast Ohio Medical University, Rootstown, USA

**Keywords:** dementia, assisted living, medication, family, Alzheimer’s disease

## Abstract

Antipsychotic medications are frequently prescribed to assisted living (AL) residents who have dementia, although there is a lack of information about the potential side effects and adverse events of these medications among this population. Oversight and monitoring by family members is an important component of AL care, and it is important to understand family awareness of antipsychotic use and reports of potential side effects and adverse events. This cross-sectional, descriptive study of family members of 283 residents with dementia receiving antipsychotic medications in 91 AL communities found high rates (93%) of symptoms that could be potential side effects and a 6% rate of potential adverse events. The majority of families were aware their relative was taking an antipsychotic. Findings suggest that obtaining family perspectives of potential side effects and adverse events related to medication use may contribute to overall improvement in the safety of AL residents living with dementia.

## Introduction

Assisted living (AL) provides housing and supportive care to more than 811,000 people in 28,900 communities in the United States (Harris-Kojetin et al., 2019). It has become the primary residential long-term care provider for people with dementia, with a recent study finding that dementia is one of the most prevalent chronic conditions (Harris-Kojetin et al., 2019), others documenting that 42% of residents have moderate/severe dementia (Ma et al., 2014), and 90% have some degree of cognitive impairment (Zimmerman et al., 2007). Dementia is associated with progressive decline that may cause behaviors such as aggression, wandering, agitation, and sleep disturbances (Peters et al., 2012). Although there are no approved medications to treat these behaviors, often they are treated by off-label use of antipsychotics (Kerns et al., 2018). Evidence on the effectiveness of antipsychotics for behaviors suggests that they are minimally effective and carry risk (Masopust et al., 2018) including sleepiness, cerebrovascular events, anticholinergic symptoms, tardive dyskinesia, and death (Ma et al., 2014). Furthermore, the potency (concentration) and prescribed high doses of antipsychotics have been associated with side effects (College of Psychiatric & Neurologic Pharmacists, 2019).

Most antipsychotic research has been conducted in general populations or nursing homes, with little focused on use in AL (Aerts et al., 2019; Hulshof et al., 2020; Van Leeuwen et al., 2018). A recent study found 57% of AL residents with behavioral expressions received medications (Zimmerman et al., 2014), and another found the use of these medications associated with poorer quality of life (Harrison et al., 2018). Absent from this and other research (Kerns et al., 2018) are perspectives of family members, who tend to remain involved in care (Port et al., 2005). Also absent is the availability of publicly reported data. This lack of a public-reporting mechanism for AL challenges research efforts examining resident outcomes (June et al., 2020; Zimmerman et al., 2013). Furthermore, because oversight and monitoring by family members is an important component of AL care (Zimmerman et al., 2018), it is important to understand family perception of resident behaviors, their awareness of antipsychotic use, and their report of symptoms that may constitute potential side effects and adverse events.

This study used a seven-state sample of AL communities, conducted extensive chart reviews, and interviewed family members to (a) examine the potency of antipsychotics used among residents living with dementia in AL and (b) based on family reports, learn their perception of potential side effects, adverse events, and knowledge of their relative’s behavior and use of medications. Results have implications to guide antipsychotic prescribing and monitoring in AL.

## Method

### Sample

AL communities from seven states (Arkansas, Louisiana, New Jersey, New York, Oklahoma, Pennsylvania, and Texas) were eligible if they were licensed to provide non-nursing services to more than four adults older than 65 years requiring long-term care (*N* = 1,624 communities). Within each state, two geographically clustered regions were constructed to represent the entire state, based on census and descriptive characteristics used in other work (Zimmerman et al., 2005). Within the regions, communities were randomly sampled proportionate to bed size, with an aim to enroll 35 to 40 AL communities per state. To avoid historic effects of prescribing patterns over time, one half of the data were collected in each state first, and then again in the second half (October 2016–November 2018). During recruitment, 743 communities were invited to participate; 354 (48%) refused, nine (1%) provided partial data, and 130 (17%) had a pending recruitment status at study end; in total, 250 participated. Participating and nonparticipating communities did not differ by size (*p* = .43). Families of 494 residents from 117 communities were invited to participate in the second round of data collection; their data and those of their relatives are included in these analyses.

### Data Collection

Two data sources are used in these analyses. In each AL community, up to 15 charts were randomly reviewed of residents who had dementia and were taking an antipsychotic medication; data included demographics, medications, and diagnoses. The second source of data was telephone interviews with a family member of the same residents, conducted within 4 weeks of chart abstraction. A Health Insurance Portability and Accountability Act (HIPAA) waiver was obtained to abstract chart information, and family members provided informed consent prior to the interview. Data collectors were blinded to sample selection. All procedures were approved by the Office of Human Research Ethics of the University of North Carolina at Chapel Hill.

### Measures

Use of antipsychotic medications was defined as receipt of any type, form, or dose of any antipsychotic within the 7 days prior to data collection. A 7-day lookback was chosen because the medications of interest are taken at least weekly. Antipsychotics included first-generation/typical and second-generation/atypical agents. These and other data (e.g., diagnoses) were obtained from AL records. Family interviews were conducted using a structured interview guide comprising closed-ended questions and standardized scales. Family reported on presence of symptoms that could be potential medication side effects by responding to a list of symptoms related to anticholinergic effects (constipation or dry mouth); psychomotor function (akinesia [lack of movement], akathisia [constant movement], rigidity when walking, and tardive dyskinesia [uncontrollable movements]); and neurological/psychological effects (anxiety, falling asleep during the day, dizziness when standing, and apathy). They also reported on potential adverse events, including seizures, heart attack/myocardial infarction, stroke/transient ischemic attack, hip fracture, or 10-lb weight loss or gain over the previous 3 months. In addition, families were asked standardized questions about whether the resident had ever required care for behaviors such as agitation or aggression or had received medications for those behaviors while an AL resident. Finally, families provided information about the residents’ cognitive status using standardized scales: the Minimum Data Set Cognition Scale (MDS-COGS; Hartmaier et al., 1994), function using the Katz Index of Independence in Activities of Daily Living (ADL; Katz, 1983), and behaviors using the Cohen-Mansfield Agitation Inventory (CMAI; Cohen-Mansfield et al., 1989).

### Analysis

Descriptive characteristics (frequency, percent, means, standard deviations) were derived for all variables. Dose equivalency is an accepted method to compare antipsychotic medications (College of Psychiatric & Neurologic Pharmacists, 2019; Leucht et al., 2014, 2016), and doses were converted into a daily dose equivalence score based on a method outlined by the College of Psychiatric and Neurologic Pharmacists (College of Psychiatric & Neurologic Pharmacists, 2019). With 100 mg of chloropromazine (Thorazine) as the reference, we calculated daily dose equivalencies for the most prevalent antipsychotics: haloperidol (Haldol), perphenazine (Trilafon), aripiprazole (Abilify), olanzapine (Zyprexa), quetiapine (Seroquel), risperidone (Risperdal), and ziprasidone (Geodon). Of these, the two most frequently prescribed were compared for their associations with symptoms that could be potential side effects and potential adverse events in the prior month. Statistical significance comparing potential side effects and adverse event rates was determined using Fisher’s exact test.

## Results

Families of 494 residents from 117 AL communities were invited to participate; 283 agreed (57%), from 91 communities (Table 1 shows sample characteristics). Residents were primarily White (95.8%) women (73.4%), with an average age of 83.2 years. Family participants were primarily White (95.7%) women (64.3%) caring for a parent/parent-in-law (71.2%). The average resident had moderate to severe dementia (mean MDS-COGS score: 5.2), considerable functional impairment (mean ADL score: 3.6), and moderate agitation (mean CMAI score: 26.3). Based on chart reviews, 12% had schizophrenia and related disorders (a diagnosis associated with antipsychotic use), 46% had depression, and 33% had anxiety; families reported that 46% had anxiety. Investigating this discrepancy, we found of the 283 residents, 174 (61.5%) had either a formal, chart-based diagnosis of anxiety or a family member report of anxiousness, and 49 (28.2%) had agreement/congruence. In terms of care, 81% of families reported that staff had provided care for behaviors such as aggression, pacing, or resisting care (specifically, for any behavior included in the CMAI), and 85% reported that medication had been administered for the same behaviors.

**Table 1.** Assisted Living Resident and Family Member Characteristics (*N* = 283).

Characteristics	*N* (%) or *M* (*SD*)
Resident demographics	
Female	207 (73.4)
White	271 (95.8)
Age	83.2 (8.5)
Resident functioning (family report)	
Cognitive status (MDS-COGS; 0–10)	5.2 (2.7)
Independent functioning (Katz; 0–6)	3.6 (1.9)
Agitation behaviors (CMAI; 16–61)	26.3 (7.9)
Resident diagnoses and care	
Type of dementia	
Alzheimer’s disease	102 (36.0)
Vascular dementia	10 (3.5)
Frontotemporal dementia	1 (0.4)
Mixed dementia	1 (0.4)
Not otherwise specified/Other dementia	167 (59)
Psychiatric diagnoses	
Any^a^	184 (65.0)
Anxiety and related disorders	93 (32.9)
Bipolar and related disorders	18 (6.4)
Depression and related disorders	132 (46.4)
Personality disorder and related disorders	3 (1.1)
Schizophrenia and related disorders	34 (12.0)
Sleep disorder and related disorders	33 (11.7)
Epilepsy	14 (5.0)
Tourette’s syndrome	0 (0.0)
Parkinson’s disease	12 (4.2)
Heart disease	91 (32.2)
Cerebrovascular conditions	29 (10.3)
Chronic kidney disease	21 (7.4)
Chronic liver disease	0 (0.0)
Resident had staff providing care for behaviors such as aggression, pacing, or resisting care^b^	228 (80.6)
Resident had been given medications for behaviors such as aggression, pacing, or resisting care^b^	238 (85.0)
Family member demographics	
Family member’s relationship to resident	
Child or child-in-law	200 (71.2)
Spouse	32 (11.4)
Sibling or sibling-in-law	18 (6.4)
Other	31 (11.0)
Female	182 (64.3)
White	270 (95.7)
Age	61.4 (10.7)

*Note*. Sources = family interview and resident charts. Due to missing data, the sample size ranges from 265 to 283. MDS-COGS = Minimum Data Set Cognition Scale; Katz = Katz Index of Independence in Activities of Daily Living; CMAI = Cohen-Mansfield Agitation Inventory.

a Excludes dementia. ^b^Not mutually exclusive. The total list of behaviors were those included on the CMAI.

As shown in Table 2, quetiapine (Seroquel) and risperidone (Risperdal) were the most frequently prescribed antipsychotics, prescribed to 58% and 26% of residents, respectively. Dose equivalencies were lowest for risperidone (50 mg) and highest for ziprasidone (116.7 mg, prescribed to 1% of the sample), with those for quetiapine being more mid-range (66.7 mg). In terms of potential side effects and adverse events (Table 3), 93% had at least one potential side effect, and 19% reported five or more. The most common were neurologic/psychological effects (89% of residents) including somnolence during the day (81%), apathy (50%), and anxiety (46%). Overall, 6% of the sample reported at least one potential adverse event; 3% had seizures, 1.4% had transient ischemic attacks, and 1.4% had a hip fracture in the last month. In the previous 3 months, 18% lost more than 10 pounds, and 10% gained more than 10 pounds. When comparing symptoms for residents taking only quetiapine versus those taking only risperidone, only one comparison was significant: akinesia was reported for 18.5% of residents taking quetiapine versus 32.8% of those taking risperidone (*p* = .032).

**Table 2.** Antipsychotic Use and Dose by Medication Type (*N* = 283 Residents, 325 Medications).

Medication type and names	*N* (%) of residents taking the drug	Daily dose		Daily dose equivalence^a^
		Median (IQR)	Range	Median (IQR)
First generation/typical				
Haloperidol (Haldol)	11 (3.9)	1.3 (1.0–2.0)	0.25–8.0	65.0 (50.0–100.0)
Perphenazine (Trilafon)	1 (0.4)	4.0	4.0	50.0
Second generation/atypical				
Aripiprazole (Abilify)	8 (2.8)	5.0 (2.0–5.0)	2.0–5.0	66.7 (26.7–66.7)
Olanzapine (Zyprexa)	35 (12.4)	5.0 (2.5–10.0)	1.25–25.0	100.0 (50.0–200.0)
Quetiapine (Seroquel)	165 (58.3)	50.0 (25.0–100.0)	0.5–600.0	66.7 (33.3–133.3)
Risperidone (Risperdal)	74 (26.1)	0.5 (0.5–1.0)	0.125–6.0	50.0 (50.0–100.0)
Ziprasidone (Geodon)	4 (1.4)	70.0 (50.0–80.0)	40.0–80.0	116.7 (83.3–133.3)

*Note*. Source = resident charts. IQR = interquartile range; daily dose = strength in milligrams × daily frequency.

a Based on 100 mg of chloropromazine: haloperidol dose × 50.0 mg, perphenazine dose × 12.5 mg, aripiprazole dose × 13.3 mg; olanzapine dose × 20.0 mg, quetiapine dose ×1.3 mg, risperidone dose × 100.0 mg, ziprasidone dose × 1.6 mg (College of Psychiatric & Neurologic Pharmacists, 2019; Leucht et al., 2014, 2016).

**Table 3.** Assisted Living Resident Potential Side Effects and Adverse Events by Antipsychotic Medication (*N* = 283).

Potential side effects and adverse events	Residents taking any antipsychotic^a^ (*N* = 283), frequency (%)	Residents taking quetiapine (*n* = 153), frequency (%)	Residents taking risperidone (*n* = 64), frequency (%)	*p* value
Potential side effects in the last month				
Any	262 (92.6)	140 (91.5)	60 (93.8)	.78
1	44 (15.6)	27 (17.7)	9 (14.1)	
2	60 (21.2)	34 (22.2)	13 (20.3)	
3	53 (18.7)	27 (17.7)	11 (17.2)	
4	50 (17.7)	28 (18.3)	14 (21.9)	
5+	55 (19.4)	24 (15.7)	13 (20.3)	
Anticholinergic				
Any	65 (23.0)	34 (22.2)	15 (23.4)	.86
Was constipated	37 (13.6)	18 (12.2)	10 (16.7)	.38
Had dry mouth	39 (14.3)	21 (14.4)	9 (14.5)	1.00
Psychomotor function				
Any	150 (53.0)	75 (49.0)	40 (62.5)	.075
Hardly moved at all, tended to sit without moving (akinesia)	68 (24.3)	28 (18.5)	21 (32.8)	.032
Constantly moved, was restless, could not keep still (akathisia)	56 (20.0)	34 (22.4)	14 (21.9)	1.00
Was very stiff or rigid when moving or walking (rigidity)	58 (20.7)	29 (19.2)	12 (18.8)	1.00
Had uncontrollable, stuff, jerky movement (tardive dyskinesia)	22 (7.8)	7 (4.6)	4 (6.3)	.74
Neurological/cardiovascular/psychological side effects				
Any	251 (88.7)	134 (87.6)	57 (89.1)	.82
Was anxious (anxiety)	130 (46.1)	66 (43.1)	31 (48.4)	.55
Fell asleep during the day (somnolence)	227 (81.1)	121 (79.6)	51 (79.7)	1.00
Was dizzy when standing (dizziness)	61 (21.7)	30 (19.7)	13 (20.3)	1.00
Did not seem to care about things (apathy)	136 (49.6)	70 (47.3)	35 (55.6)	.29
Adverse events in the last month				
Any	16 (5.7)	9 (5.9)	5 (7.8)	.56
1	15 (5.3)	9 (5.9)	5 (7.8)	
2	1 (0.4)	0 (0.0)	0 (0.0)	.0
Had seizures	8 (2.8)	4 (2.6)	3 (4.7)	.42
Had a heart attack or myocardial infarction	1 (0.4)	1 (0.7)	0 (0.0)	1.00
Had a stroke or transient ischemic attack	4 (1.4)	2 (1.3)	1 (1.6)	1.00
Had a hip fracture	4 (1.4)	2 (1.3)	1 (1.6)	1.00
Weight change in the last 3 months				
Any	78 (27.6)	41 (26.8)	23 (35.9)	.19
Lost more than 10 pounds	51 (18.2)	25 (16.5)	18 (28.1)	.062
Gained more than 10 pounds	29 (10.4)	17 (11.2)	5 (7.9)	.62

*Note*. Source = family interview. The value of *p* compares residents taking only quetiapine versus those taking only risperidone using Fisher’s exact test (two-sided). Side effects and adverse events were not mutually exclusive.

a Includes haloperidol, perphenazine, aripiprazole, olanzapine, quetiapine, risperidone, and ziprasidone. Seven (2.5%) residents were taking both quetiapine and risperidone.

## Discussion

This study provided a unique opportunity to examine the potential side effects and adverse events of antipsychotic use for residents living with dementia in AL, and the potential need for family involvement in care and monitoring. Among 283 residents with dementia from 91 AL communities who were receiving antipsychotics on a regular basis, we found (a) high rates (93%) of symptoms that could be potential side effects; (b) a 6% rate of potential adverse events; (c) that quetiapine (Seroquel) and risperidone (Risperdal) were the most frequently prescribed antipsychotics; and (d) for the majority, but not all, family were aware their relative was taking an antipsychotic.

Although our research design cannot assuredly relate potential side effects and adverse events to antipsychotics, it is important to note the frequency of these symptoms and detrimental effects. Somnolence was the most frequent potential side effect (81%) and is higher than what is reported in the general population of older adults living with dementia, which ranges from 30% (Merlino et al., 2010) to 70% (Cagnin et al., 2017). Somnolence has implications for quality of life, social interaction, nutritional intake, and function (Lee et al., 2007); it could suggest a potential problem with medication tolerance requiring dose adjustment and/or need for nonpharmacological interventions (Ma et al., 2014). After somnolence, families reported apathy (50%) and anxiety (46%) as most common. In the general population of people living with dementia, rates of apathy range from 49% to 61% (Clarke et al., 2008), and anxiety ranges from 39% (Zhao et al., 2016) to 46% (Ferretti et al., 2001), suggesting that the rates reported in this study are comparable. However, family reports of anxiety matched staff reports only 28% of the time, indicating a need for better recognition of potential side effects and adverse events on the part of both parties. Previous research on apathy (Harrison et al., 2016) and anxiety (Badrakalimuthu & Tarbuck, 2012) in people with dementia suggests that a reduction of antipsychotics paired with nonpharmacological interventions can significantly reduce these symptoms (Badrakalimuthu & Tarbuck, 2012; Harrison et al., 2016).

The overall rate of any potential adverse event was 6%, with seizures being most common (3%). Although seizures can have multiple causes (including dementia), previous research on seizures and antipsychotics found that people with dementia had a significantly higher incidence of first-time seizures than people with affective disorders (Bloechliger et al., 2015), attesting to the serious implications of antipsychotic use. Also, 28% reported a weight change in the last 3 months; weight loss (18%) predicts mortality in people with dementia (White et al., 1998), and weight gain (10%) can lead to conditions such as diabetes and hyperlipidemia (Ames et al., 2016). In general, any major change in weight, especially over a short period, is a cause for concern. The potential adverse event findings from this study point to the need for AL residents living with dementia to be closely monitored by staff and families.

The dose equivalency findings indicate a wide range of exposure that may suggest increased risk of adverse outcomes. Previous research found a 3.5% greater mortality risk in persons with dementia receiving high doses of quetiapine, olanzapine, and risperidone than those receiving lower doses (Maust et al., 2015). Our findings suggest that clinicians should consider not only the general risks associated with prescribing antipsychotics but also the dose and dose equivalency to prevent harm.

Another important finding is that 81% of families reported that staff had provided care for behaviors, and 85% reported that medication had been administered for the same behaviors (meaning that only 15% were unaware their relative was taking a medication). Because families of AL residents play a role in monitoring their relative’s medical status and well-being (Port et al., 2005; Tjia et al., 2017), our findings suggest monitoring should extend to behaviors and include awareness of medications, potential side effects, and adverse events. Given the potential seriousness of these side effects/adverse events, it is important to consider how and in what ways families are involved in care. It could be that more purposeful contact with residents’ families—including discussing their perspectives of potential side effects and adverse events related to medication use—may identify changes in resident status that could contribute to overall improvement in the safety of AL residents living with dementia.

It is important to recognize, however, that our sample included residents living with dementia who had a family caregiver; other research has shown that family contact after placement into long-term care may be tied to socioeconomic factors—family involvement is more common for residents not on Medicaid, with close-knit families and more proximate social network, and who are White (Gaugler et al., 2004; Port et al., 2001). Consequently, more concerted efforts may be needed to alleviate disparities for residents who are more financially and socially disadvantaged and of minority status (our sample was 95.8% White). It is possible that their experience with potential side effects and adverse events may be different than residents who have family and are from other racial backgrounds. Current research recognizes the variability of family involvement in the antipsychotic prescribing process (Tjia et al., 2017) and that resident characteristics such as race (Kales et al., 2007, 2012) may be related to antipsychotic medication use and outcomes. Future research examining how resident characteristics relate to outcomes is an important line of inquiry to address potential health and racial disparities in AL.

This study reports novel findings and provides new information about AL residents receiving antipsychotics, but has limitations. First, the study sample is not necessarily representative of all AL communities in the United States. Second, it cannot be claimed that the potential side effects and adverse events examined were caused by the antipsychotics. That said, the mere prevalence of these potential side effects and adverse events is important when considering optimal care. Future efforts should examine in more detail the causes of these potential side effects and adverse events.

This study has important implications for improving care for AL residents living with dementia, including the importance of meeting the physical and psychological needs of these individuals when they exhibit behaviors indicating distress. Of note, subpopulations, such as people living with Lewy Body Dementia, have been known to have catastrophic outcomes when antipsychotic medications are administered (Tampi et al., 2016). Alternatively, antipsychotic medications can be effective in treating psychosis, aggression, and agitation in people living with dementia (Tampi et al., 2016; Van Leeuwen et al., 2018). Thus, antipsychotic medications should be explored further to delineate the risks and benefits and types of dementia that are most appropriate for use.

It is important to consider these findings within the context of AL—a long-term care setting that provides housing and supportive care. AL communities vary by size and services; some provide care to a small number of residents primarily by personal care aides who have minimal medical or nursing oversight, and others provide care to residents in large communities with full-time nursing staff and medical directors (Beeber et al., 2018). This variation extends to the ability to oversee pharmacological management of behaviors and symptoms. While clinicians should always practice caution when prescribing antipsychotics to older adults with dementia, they also should be aware that patients who reside in AL may not be receiving skilled medical oversight. In particular, research on medication management in AL has focused largely on training needed for medication administration and preventing and reducing medication errors (Carder, 2017; Carder & O’Keeffe, 2016; Zimmerman et al., 2011). Given the findings from this study, care should extend to monitoring AL residents for potential side effects and adverse events related to antipsychotic medications.
